# Plant Leucine-Rich Repeat Receptor Kinase (LRR-RK): Structure, Ligand Perception, and Activation Mechanism

**DOI:** 10.3390/molecules24173081

**Published:** 2019-08-25

**Authors:** Sayan Chakraborty, Brian Nguyen, Syed Danyal Wasti, Guozhou Xu

**Affiliations:** Department of Molecular and Structural Biochemistry, North Carolina State University, Raleigh, NC 27695, USA

**Keywords:** receptor kinase, LRR-RK, signaling, X-ray crystallography

## Abstract

In recent years, secreted peptides have been recognized as essential mediators of intercellular communication which governs plant growth, development, environmental interactions, and other mediated biological responses, such as stem cell homeostasis, cell proliferation, wound healing, hormone sensation, immune defense, and symbiosis, among others. Many of the known secreted peptide ligand receptors belong to the leucine-rich repeat receptor kinase (LRR-RK) family of membrane integral receptors, which contain more than 200 members within *Arabidopsis* making it the largest family of plant receptor kinases (RKs). Genetic and biochemical studies have provided valuable data regarding peptide ligands and LRR-RKs, however, visualization of ligand/LRR-RK complex structures at the atomic level is vital to understand the functions of LRR-RKs and their mediated biological processes. The structures of many plant LRR-RK receptors in complex with corresponding ligands have been solved by X-ray crystallography, revealing new mechanisms of ligand-induced receptor kinase activation. In this review, we briefly elaborate the peptide ligands, and aim to detail the structures and mechanisms of LRR-RK activation as induced by secreted peptide ligands within plants.

## 1. Introduction

Plant development is mostly a post-embryonic event in which intercellular signaling and cell-cell communication play a large role [1]. In recent years, secreted peptides have been considered as an essential component of intercellular communication that facilitates plant growth, development, interactions with the environment, and other biological responses [1]. Membrane integral receptor kinases (RKs) are necessary for the plant intercellular signaling network as they perceive these secreted peptides and allow plants to respond to various external and internal cues to regulate their growth and development. Bioinformatics analysis has predicted the presence of around 600 RKs within the *Arabidopsis* genome [2]. Many of these RKs play critical roles in plant growth, development, defense, reproduction, and symbiosis [3]. Ligand binding presumably induces the dimerization or oligomerization of RKs with either themselves or with a co-receptor, leading to the activation of an intracellular kinase domains (KD) to initiate downstream signaling transduction and regulate multiple biological functions [4,5].

Based on their ectodomain structures, plant RKs can be classified into 14 subfamilies [2,6]. The leucine-rich repeat receptor kinase (LRR-RK) family of membrane integral receptors which contains more than 200 members in *Arabidopsis* is considered to be the largest family of plant receptor kinases [6,7]. The LRR-RKs are composed of an extracellular domain containing the consensus hydrophobic leucine residues, a single membrane-spanning domain, and a cytoplasmic kinase domain with serine/threonine specificity [7]. LRR-RKs perceive different peptide ligands, hormones, and can control multiple cellular processes such as cell division, proliferation, differentiation, stem cell balance, etc. Some of the LRR-RKs function as heterodimers that form receptor complexes with other Leucine Rich Repeat-Receptor Like Proteins (LRR-RLPs). LRR-RLPs are similar to LRR-RKs in structure but lack the cytoplasmic kinase domain [8]. Both LRR-RKs and LRR-RLPs have been found to play significant roles in plant development and immunity [9].

Apart from LRR-RKs and LRR-RLPs, another class of LRR containing proteins is present in plant cells known as LRR-extensins (LRXs) which are localized within the cell wall. The *Arabidopsis thaliana* genome is predicted to encode 11 LRXs [10]. Structurally, LRXs can be recognized by a short N-terminal domain containing approximately 10 LRRs, a cysteine-rich motif, and a C-terminal extensin like domain consisting of multiple pentapeptide repeats with serines and hydroxyprolines. Certain LRXs, such as LRX1 of *Arabidopsis* are expected to be involved in regulating proper root hair cell morphogenesis and elongation [11].

LRR-RKs have been studied extensively with a large quantity of data concerning peptide ligands and LRR-RKs having been obtained from genetic and biochemical experiments [12,13,14,15] (Table 1). However, visualization of ligand/LRR-RK complex structures at the atomic level is also necessary to understand the mechanism of LRR-RKs mediated biological processes. Many LRR-RK ectodomain structures have been reported in recent years, providing insight on modes of ligand binding, structural rearrangements, heterodimerization, as well as the mechanisms of receptor activation [16,17,18,19]. This review aims to highlight the structural characterization of multiple LRR-RK ectodomains and their complexes with corresponding peptide ligands and co-receptors that have broadened our knowledge about plant receptor kinase activation.

## 2. Secreted Peptide Ligand/Hormones

Eukaryotes use a variety of peptides and steroids to mediate intercellular communication. In mammals, peptides act as hormones and growth factors that take part in multiple signaling pathways to regulate various cellular processes [66]. In plants, well-known hormones such as auxin, cytokinins, brassinosteroids, and others are responsible for governing various cellular communication pathways. The contribution of these phytohormones in plant signaling is well documented. The first plant peptide hormone systemin was identified in 1994 [67]. Systemin, an 18 amino acid long peptide, plays a role in plant cell-cell communication [67]. Systemin was found to be involved in both plant defense and wound response, as well as biotic and abiotic stress responses [67,68]. After the discovery of systemin, extensive studies have identified many other plant peptide hormones such as phytosulfokine (PSK), LURE, CLAVATA3 (CLV3), etc. [69,70,71]. In *Arabidopsis thaliana*, studies predict that more than 1000 genes may encode secreted peptides [72]. These findings suggest that similar to other eukaryotes, plants also employ peptide hormones as intercellular signaling molecules.

Most of the plant LRR-RLK ligands are part of either paracrine or autocrine signaling mechanisms. In the case of the paracrine signaling system, the diffusible molecule released from one cell can act on neighboring target cells. The best example of paracrine signaling system is the CLV3 ligand, which is expressed in the central zone of shoot apical meristem (SAM), and can exert its effects within a short range of cells [7]. In the case of PSK, which is part of the autocrine signal transduction system, both the receptor PSKR and the ligand are expressed by the same cell where the ligand activates the receptor [73]. Since most of these ligands are diffusible molecules, they can influence both nearby cells and long-distance targets [73].

Within plants, secreted peptides act extracellularly and are recognized by the transmembrane receptors present on the neighboring cells and can regulate cellular signaling pathways. Previously plant secreted peptides were classified into two major groups: small post-translationally modified peptides and cysteine-rich peptides (CRPs). Mature post-translationally modified peptides are smaller, ranging from 5 to 20 amino acids [74]. On the other hand, CRPs are relatively larger with 50–100 amino acids [75,76]. CRPs usually contain an even number of cysteine residues that form intramolecular disulfide bonds.

A majority of the plant peptides are derived from a non-functional pre-protein that both gets proteolytically processed and/or enzymatically modified to produce biologically active peptides [77]. However, recent studies have shown that functionally active precursor proteins can also produce peptides that can have different activity than the mother protein [78,79,80]. Reports have also shown that short open reading frames (sORF) present in the 5’ leading strand of a mRNA can be translated into small peptides with no precursor protein necessary for their synthesis [81,82,83]. Due to the increasing complexity of plant peptidome and after the identification of various different novel types of peptides, Tavormina et al. have come up with a comprehensive peptide classification [77]. Based on their classification, plant peptides can be grouped into three major clades: (a) peptides that originated from a non-functional precursor, (b) peptides that originated from functional precursors, and (c) peptides not produced from precursor proteins [77].

In this review, we will focus mainly on the peptide ligands and their corresponding LRR-RLKs that have been structurally characterized.

### 2.1. Post-Translationally Modified Peptides

**Figure 1:**
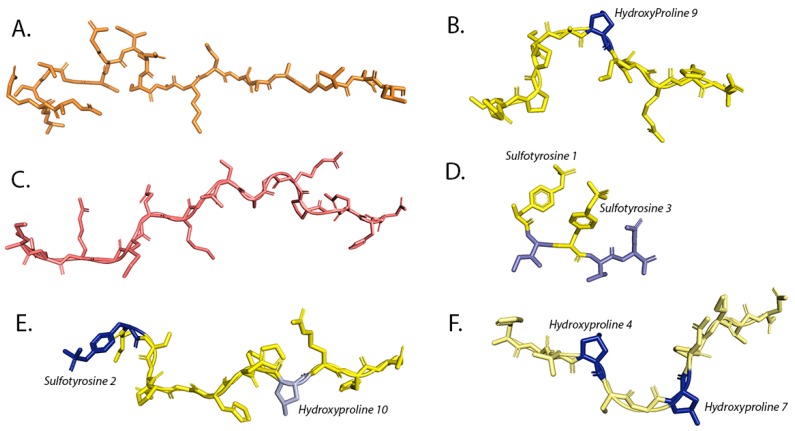
Structures of small secreted peptides ligands of LRR-RLKs in stick representation: flg22, Inflorescence Deficient in Abscission (IDA), PEP1, phytosulfokine (PSK), RGF1, and tracheary element differentiation inhibitory factor (TDIF). (**A**) flg22 (colored in orange) is the epitope of bacterial flagellin that can elicit immune response after interacting with FLS2 ectodomain (PDB ID 4MNA). (**B**) IDA (colored in yellow) interacts with HAESA LRR ectodomain (PDB ID: 5IXQ). IDA contains a hydroxyproline residue at the ninth position (colored in blue) (**C**) PEP1 peptide (colored in salmon) is responsible for generating immune response in the plant by inducing heterodimerization of PEPR1 (PDB ID 5GR8) and its co-receptor BAK1. (**D**) PSK is a five amino acid long hormone (colored in cyan) that contains two sulfo-tyrosine residues (colored in yellow). These two sulfate moieties directly interact with the PSKR LRR (PDB ID: 4Z63). (**E**) RGF1 peptide (colored in yellow) is involved in maintaining stem cell niche in root and is perceived by the RGFR1 receptor. (PDB ID 5HYX). RGF1 peptide contains two post-translationally modified residues; sulfated tyrosine (colored in blue) and hydroxyproline (colored in cyan). (**F**) TDIF is a dodecapeptide (colored in pale yellow) which is perceived by the TDR receptor (PDB ID 5JFI). TDIF inhibits xylem cell differentiation and promotes procambial cell proliferation. It contains two hydroxyproline residues in fourth and seventh position (colored in blue).

Proteolytic processing is an integral step in producing small post-translationally modified peptides. This process involves a large precursor polypeptide containing an N-terminal signaling sequence which is translated and subsequently cleaved by proteases and modified to yield a smaller mature functional peptide. Major post-translational modifications that occur on these peptides include sulfation of tyrosine, hydroxylation of proline, and arabinosylation in plants [84]. Hydroxylation of proline is a common modification catalyzed by Prolyl-4-Hydroxylase (P4H) enzyme that has been found in most known small mature modified plant peptides except for PSK [85] (Figure 1). There is currently no recognition motif that has been identified for proline hydroxylation [86]. Sulfation of tyrosine modification has been discerned in PSK, plant peptide containing sulfated tyrosine (PSY), Casparian Strip Integrity Factors (CIFs), and Root Meristem growth Factor (RGF) peptides [37,69,87] (Figure 1). Tyrosine modification is catalyzed by Tyrosyl Protein Sulfotransferase (TPST) enzymes [88]. Although the consensus sequence of tyrosine sulfation is unclear, it has been shown that the presence of aspartic acid, and other acidic amino acids near the tyrosine, significantly improves the chance of sulfation [89]. In this section we will focus mostly on the CLE peptides and will briefly describe some of the other important post-translationally modified peptides.

The hydroxyproline residues of peptides like CLV3, CLE9, and CLE2 are further modified by the addition of L-Arabinose sugar moiety [43,86,90]. Hydroxyproline O-arabinosyltransferase (HPAT) enzyme is responsible for this arabinosylation reaction. It is believed that arabinosylated peptides play crucial roles within plant growth, development, and defense [43].

CLAVATA3 (CLV3)/endosperm surrounding region (CLE) family of peptides is one of the most studied families of small post-translationally modified peptides. In *Arabidopsis*, the CLE family of small secretory proteins can be processed to 12–13 amino acid residue peptides with two conserved proline residues at positions four and seven that are usually hydroxylated [91]. In the case of CLV3 peptide, the hydroxyproline at the seventh position is arabinosylated to form a tri-arabinose chain [43]. Whether hydroxylation is required for receptor binding of CLE peptides and their activation is still controversial; however, the arabinosylation of CLV3 is known to be necessary for its in vivo function. The CLE peptides are encoded by a family of 32 *CLE* genes, including *CLV3*, which express proteins smaller than 15 kD [1,91,92]. Along with the N-terminal signal peptide (SP) motif, CLE proteins contain a large portion of amino acid sequences in the middle that have no conservation within the family.

In contrast, the CLE domain located at the C-terminus is highly conserved. Intriguingly, the middle non-conserved region is mostly dispensable for their in vivo function [93]. It was found that chemically synthesized 12 amino acid residue peptides corresponding to the CLE domain are functionally active when applied to plants [94,95,96]. When 22 synthetic peptides, encoded by 26 *CLE* genes, were applied to *Arabidopsis* seedlings, 18 of them were shown to arrest root growth. These 18 synthetic peptides include CLV3 and are classified as A-type. The remaining four peptides, CLE41p, CLE42p, CLE43p, and CLE44p do not influence root growth and are classified as B-type [95].

Studies of these two groups have shown that A-type CLE peptides function to define the balance between stem cell proliferation and differentiation in Shoot Apical Meristem (SAM) or Root Apical Meristem (RAM) [42,93,94], while the B-type CLE peptides function to promote the development of vasculature procambial cells and suppress xylem differentiation without inducing the consumption of SAM or RAM [45,96]. Interestingly, some A-type CLE peptides can act synergistically with B-type peptides to stimulate vascular cell proliferation [95]. In B-type CLE peptides, CLE41 and CLE44 both encode the same CLE peptide tracheary element differentiation inhibitory factor (TDIF) [45].

*Arabidopsis*, soybean, and common bean contain 32, 84, and 44 *CLE* peptide-encoding genes respectively [97]. Bioinformatics-based phylogenetic analysis on the CLE pre-pro-peptides from soybean, common bean and *Arabidopsis* show that the pre-pro-peptide CLEs could be grouped based on their functions and CLE domains. The CLE pre-pro-peptides were classified into seven distinct groups (Groups I–VII) [97]. This new classification is an expansion of the A and B type grouping of CLE peptides in *Arabidopsis* [98]. In this new grouping system, both CLE40 and CLV3 belong to group I, TDIF is placed in group III, and group VI comprises rhizobia induced CLE peptides (RICs) [97].

The LRR-RK protein CLVAVATA1 (CLV1) has been identified as the plasma membrane receptor for CLV3 [13,14,71,99]. Direct interaction between the CLV3 peptide and the ectodomain of CLV1 has been confirmed by genetic studies [13]. It has been reported that the arabinosylation on the seventh hydroxyproline elevates the binding affinity of a 13-residue glycol-CLV3 peptide to the CLV1 receptor and its bioactivity [43,100]. In addition to CLV1, the receptor protein kinase 2, CORYNE, and the LRR receptor-like protein CLV2 have also been shown to interact with CLV3 to regulate SAM development [101,102,103]. Furthermore, three CLV1-related receptors known as BARELY ANY MERISTEM 1, 2, and 3 (BAM1, 2, and 3) also play a similar biochemical role to perceive CLV3 when ectopically expressed in SAM [47,48,104]. CLV3 is secreted by the apical stem cells that stay on the upper portion of SAM. Perception of CLV3 in the organization center (OC) limits the expression of the homeodomain transcription factor WUSCHEL (WUS). WUS is known to be responsible for maintaining the stem cell fate of meristem. CLV3-WUS forms a feedback loop, with WUS controlling the expression of CLV3 and CLV3 in turn restricting WUS expression and distribution in the middle of the OC. This feedback regulation ensures the delicate balance between meristem stem cell proliferation and differentiation, a crucial aspect for plant growth and organ development [91]. The downstream signaling events after the CLV3/receptors interactions are not well understood. It has been indicated that a MAPK cascade is engaged upon receptor activation [105]. Furthermore, a protein phosphatase KAPP and a Rho-like GTPase ROP have been shown to play a regulatory role in signaling [99], while two more phosphatases POL and PLL1 may also act downstream in the pathway to activate WUS [106].

Two *CLE* genes *CLE41* and *CLE44* both encode the same B-type CLE peptide TDIF, which is hydroxylated and proteolytically processed to a 12 amino acid residue peptide TDIF [45] (Figure 1). TDR(PXY) is the receptor for TDIF peptide that is also a LRR-RK that shares 42% sequence identity with CLV1. Structural, biochemical, and genetic evidence has confirmed the specific physical interaction between the ligand and the receptor [45,107]. Phloem cells secrete TDIF that then acts on vascular procambial cells to promote self-proliferation while also suppressing xylem differentiation. A WUSCHEL-related HOMEOBOX 4 transcription factor (WOX4) is a downstream target of TDIF/TDR that leads to cell proliferation but not to xylem differentiation [108]. Besides, members of GSK3, BIN1, SKI, and SKII mediate the regulation of a transcription factor BES1 to influence xylem differentiation but not in procambial cell proliferation [109]. Besides CLV3 and TDIF, membrane receptors for most other CLE peptides have yet to be identified, with the in vivo functions of their genes remaining to be elucidated.

PSKs, PSYs, RGFs, and CIFs are the four major peptide families that require sulfation on their tyrosine residues for their functional activity. PSK is a di-sulfated penta-peptide and is derived from a non-functional precursor protein [69]. In *Arabidopsis*, PSK is perceived by an LRR-RK known as PSKR1 [32]. It has been noted that PSKR2 can also recognize PSK but to a lesser extent [33,110]. It is involved in multiple activities such as cell division, proliferation, induction of root growth, as well as plant pathogen interactions [34,69,110]. PSY1 belongs to another group of sulfated peptides that are 18 amino acids long and also contains a tri-arabinose sugar moiety anchored at one of the two hydroxyl residues [37]. PSY1 originated from a 75 amino acid long non-functional protein precursor [37]. PSY1R is the cognate LRR receptor for PSY1 peptide that helps in promoting cell proliferation, expansion and also involved in plant immune response generation [35,37]. In the case of *Arabidopsis*, PSY1 ligand binding phosphorylates PSY1R receptor and in turn activates AHA1 and AHA2, the proton pumps present in the plasma membrane [111]. PSKR also utilizes AHA1 and AHA2 to propagate the signaling cascade [112].

Casparian Strip Integrity Factors, CIF1/2 peptides are expressed in the root and are perceived by GSO1 and GSO2 LRR-RKs [63,113]. The casparian strip network in the root acts as a major diffusion barrier and plays an important role in plant nutrition [114]. CIF peptides are also derived from an 80 amino acid long inactive precursor protein molecule [115]. Root Meristem growth Factor (RGF) peptides, also known as GOLVEN and CLE-like (CLEL), and their actions are also very similar as they are involved in post-embryonic root development and play an important role in maintaining root stem cell niche [87,116,117]. RGF peptides vary in length, around 13–18 amino acids long. They contain two different post-translational modifications, sulfation of the tyrosine residues, and hydroxylation of the proline residues [87,117,118]. It has been found that sulfation of these tyrosine residues is important for its bioactivity [87,116,117,118]. The root meristem development is regulated by RGF peptides via two transcription factors PLETHORA1 (PLT1) and PLT2 [87]. So far, five RGF receptors have been identified that participate in RGF mediated signaling pathway [118,119,120]. Multiple proteomics-based studies on higher order plants like *Zea mays*, *Glycine max*, and *Brassica* have shown the presence of proteins in xylem sap [121,122,123,124,125]. Xylem Sap Associated Peptides (XAP) detected in *Medicago Truncatula* have been found to inhibit lateral growth of the root [125]. A total of 46 XAP peptides have been identified in *Medicago*, soybean, and *Arabidopsis* species [125]. It has been found that the N-terminal region of XAP peptides are quite conserved, whereas the C-terminal region is mostly variable and these XAP peptides contain a conserved DY sulfation motif [125]. Moreover, due to the variability in the C-terminal region, mature XAP peptides have different lengths [90,125,126]. These XAPs are also considered to be long-distance signaling peptides, thereby implying that secreted plant peptides do not always target just nearby cells [90,126].

Another peptide, Inflorescence Deficient in Abscission (IDA) also originates from a proteolytically cleaved non-functional precursor [127]. IDA peptide is an integral part of the plant floral organ shedding process [128,129,130]. In Arabidopsis, IDA peptides are expressed in various tissues like floral tissue abscission zone (AZ), root tip, and pedicel base etc. [127,130,131]. Moreover, IDA peptides and IDA Like (IDL) peptides are highly conserved across the plant species [130,132]. These peptides contain a proline residue in position nine that can be post-translationally modified to hydroxyproline [130,132]. IDA peptides interact with an LRR-RK, known as HAESA to trigger the floral abscission signaling pathway [127,133]. The C-terminal region of IDA peptides are fairly conserved and contain a motif known as PIP motif [133]. Structural and biochemical studies have shown that this PIP motif is capable of interacting with the HAESA ectodomain [133].

### 2.2. Cysteine-Rich Peptides (CRPs)

**Figure 2:**
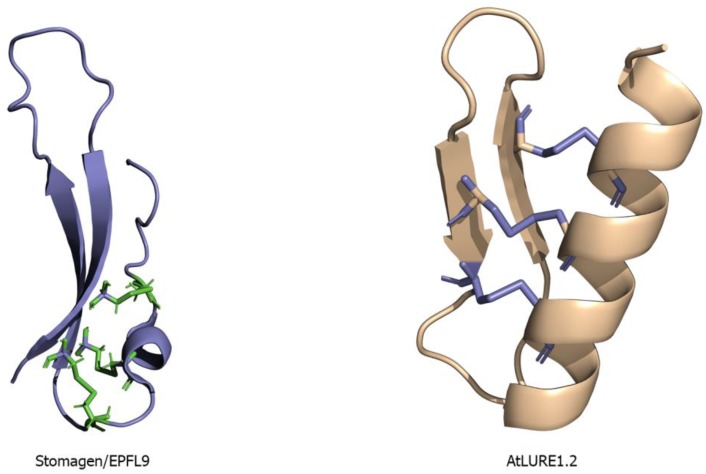
Structural representation of two cysteine-rich peptides (CRPs): Left panel, ribbon diagram showing the NMR structure of Stomagen (EPFL9) (PDB ID 2LIY) with cysteine residues shown in green colored stick representation. Stomagen is responsible for regulating the stomatal density on leaves. Right panel, ribbon diagram showing the crystal structure of AtLURE1.2 (PDB ID 5Y9W) peptide with cysteine residues represented by stick representation and colored in slate. AtLURE1.2 peptide is important for pollen tube development and ovule targeting.

CRPs can be characterized by the presence of a conserved N-terminal region that includes a secretion signal sequence and a C-terminal cysteine-rich region possessing 4–16 cysteine residues. Some CRPs like RALFs and STOMAGEN require to be proteolytically processed to produce the mature, biologically active peptides [66,134] (Figure 2). CRPs are involved in various biological pathways within plants such as plant defense, pathogen recognition, plant development, stomatal patterning, pollen tube growth, and plant reproduction [25,135]. The intramolecular di-sulfide bonding is important for proper folding and functionality [134,136]. Multiple CRPs have been discovered and characterized over the years, like Plant DEFENSINS, Nonspecific Lipid Transfer Proteins (nsLTPs), KNOTTIN-type, Cyclotides, Embryo Surrounding Factors (ESFs) etc. [137,138,139,140,141]. In this section we will concentrate mostly on Epidermal Patterning Factors Like (EPFL) and LURE peptides.

During stomatal development, the “one-cell-spacing rule” is enforced by short distance communications between neighboring cells, which is governed by the specific interactions between the secreted EPFL family cysteine-rich proteins, as well as the ERECTA family of LRR domain-containing transmembrane receptors (ERfs) [142,143]. Both EPF1 and EPF2 belong to the EPFL family, which is composed of 11 members, all of which encode cysteine-rich secretory proteins that regulate the number and position of epidermal cells forming the stomata [134,144]. Both EPF1 and EPF2 are recognized by the ERfs, which consist of ERECTA, ERL1, and ERL2. An LRR-RLP TMM, which lacks a cytoplasmic kinase domain, is also required for the stomatal fate determination [145,146]. It is generally thought that EPFL-ERfs/TMM interactions activate a downstream MAPK signaling cascade, which contains YODA as a MAPKKK, MKK4/5 as the MAPKKs, and MPK3/6 as the MAPKs [147,148,149]. The activation of the MAPK cascade will eventually lead to the phosphorylation of the bHLH family of transcription factors such as SPCH, MUTE, and FAMA that form heterodimers with either SCRM (SCREAM) or SCRM2. These bHLH transcription factors control the sequential cell fate switches in stomatal development, with phosphorylation imposed by upstream signaling inhibiting their functions and leading to the suppression of stomatal development of cells [150,151,152,153,154,155,156,157,158,159,160,161].

DEFENSIN peptides also belong to the CRPs and have been found to resist a variety of plant pathogens. These antimicrobial peptides are small (5–6 kDa) in size with more than 300 DEFENSIN peptides being identified within *Medicago* or *Arabiodpsis* [75,162]. In *Arabidopsis*, these peptides can act either in a tissue-specific manner, induced by pathogens or wound response. In peas, two genes encode two CRPs, ENOD3 and ENOD14 [163]. These CRPs are not involved in pathogen resistance and the antimicrobial response but are essential for a plant-bacterial symbiotic relationship. Several CRPs have been identified in the roots of different plants. RALF was first identified in tobacco and was later found in sugarcane and *Arabidopsis* [164,165,166]. In vitro experiments have shown that RALF peptides are essential for root development. RALF peptides harbor four conserved cysteine residues forming two disulfide bonds that are essential for the correct conformation of the peptides. It has been shown that deletion of these disulfide bonds interferes with the proper structure of the peptide, making it inactive [80]. It is also believed that RALF activity is receptor-mediated with the peptide taking an active role in the MAPK signaling cascade.

In tomatoes, some CRPs such as LAT52, LeSTIG1 have been identified to play important roles in pollen tube germination, pollen tube elongation, etc. LAT52 interacts with two specific pollen receptor kinases LePRK1 and LePRK2 [167,168]. Another group of DEFENSIN like peptides, LUREs, have been identified as pollen tube attractants. LURE1 and LURE2 were first identified in *Torenia fourneri* and were shown to be involved in micropylar pollen tube guidance. Mature LUREs from *Torenia* species are 62–70 amino acids long and contain six cysteine residues [70] (Figure 2). Recently in *Arabidopsis thaliana*, five LURE peptides (AtLURE1.1–AtLURE1.5) have been identified as attractants to regulate pollen tube guidance. It has been shown that all AtLURE peptides are capable of attracting pollen tube except for AtLURE1.5 which has only five cysteine residues instead of six [169]. Pollen Receptor Kinase 3 (AtPRK3), together with AtPRK1, AtPRK6, and AtPRK8 have been identified in *Arabidopsis thaliana* as the cell surface receptors to perceive AtLURE1.1 and AtLURE1.2 signaling molecules [53]. Additionally, Male Discoverer1 (MDIS1), MDIS1-Interacting Receptor-Like Kinase1 (MIK1), and MIK2 from *Arabidopsis* have been implicated in the LURE-mediated signaling pathway [46].

Recently, XIUQIU cysteine rich peptides have been identified, which can attract pollen tubes in a non-species-specific way [170]. XIUQIU peptides are similar to AtLURE1 peptides and are conserved in various Brassicaceae species [170]. These newly found peptides are also secreted from synergid cells and diffuse to reach the surface of the placenta [170]. It has been demonstrated that all four recombinantly expressed XIUQIU peptides can attract pollen tubes from both *Arabidopsis thaliana*, *Arabidopsis helleri,* and *Arabidopsis lyrata*. These peptides are capable of attracting *prk6* mutated pollen tubes, implying that their signaling cascade does not require PRK6 as a receptor [170]. However, the receptor kinases required for the XIUQIU peptide signaling are still unknown.

## 3. Structural Characterization of LRR-RK Ectodomain

### 3.1. Consensus Sequence and Architecture

Bacteria, yeast, animals, and humans all contain proteins with LRR motifs [171]. Animals have many cell surface receptors with LRR ectodomains [172]. In humans, the LRR motif was first discovered in a protein known as Leucine Rich α2-glycoprotein [173]. Human membrane-bound Toll-like receptors (TLRs) have a large LRR extracellular domain which has been studied in molecular detail [174,175,176,177]. It has been found that in animals and bacteria, the LRR motifs usually form a horseshoe-shaped structure [172]. While in plants, the LRR domains commonly fold in a large superhelical, S-shaped twisted structure [16,17,18]. Based on LRR structural models, it has been demonstrated that β-turn/β-sheet structures when arranged in tandem, can constitute a surface suitable for protein-protein interaction [171]. In plants, the LRR motif was first discovered in a bean polygalacturonase-inhibiting protein (PGIP) [178]. PGIP contains 10 LRRs that exhibit the characteristic plant-specific superhelical architecture. Later the LRR motif was also found in the Transmembrane Kinase 1 (TMK1) of *Arabidopsis* and other LRR-RKs [179].

**Figure 3:**
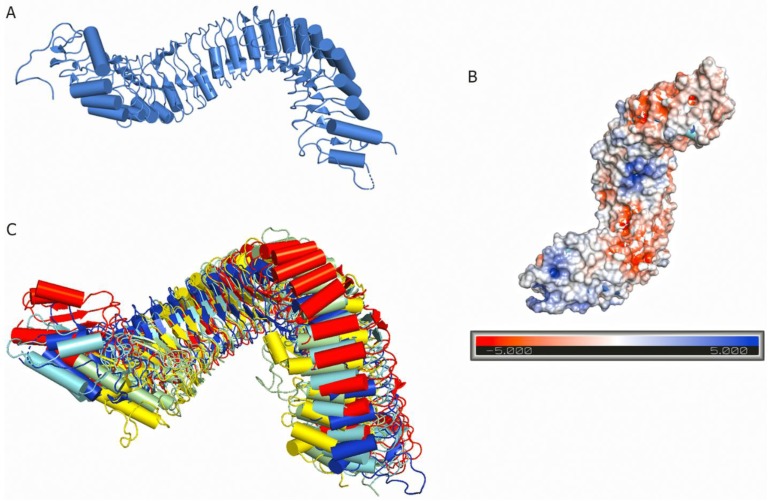
Structure of an LRR domain with a twisted superhelical assembly: (**A**) Ribbon diagram showing the superhelical arrangement of the TDR receptor ectodomain (PDB ID 5JFK) colored in blue. (**B**) The TDR receptor ectodomain is shown in a surface model colored by electrostatic potential. Blue color denotes positively charged surface patches; red means negatively charged surface; and white is neutral surface regions. (**C**) Structural superposition of five different LRR ectodomains; FLS2 is shown in red (PDB ID 4MNA), BRI1 in pale-green (PDB ID: 3RGZ), TDR is in blue (PDB ID 5JFK), PSKR1 in yellow (PDB ID: 4Z63), and PEPR1 (PDB ID 5GR8) is denoted by cyan.

Plant LRR-RK ectodomains are made up of 1–30 individual leucine-rich repeats; each repeat is generally 20–29 amino acids long [7]. The plant LRR motif has a consensus sequence xxLxxLxxLxxLxLxxNxLt/sGxIP where x stands for any amino acid, and the denoted leucine residues may sometimes be replaced with other hydrophobic residues such as valine or isoleucine [7]. The GxIP motif is unique to plant LRR-RKs, and it is widely believed that the GxIP motif is responsible for the twisted superhelical assembly of plant LRRs [180] (Figure 3). In the LRR ectodomain structure, the conserved leucine residues provide a structural backbone, whereas some variable non-conserved residues are important for protein-protein interactions [172,181].

**Figure 4:**
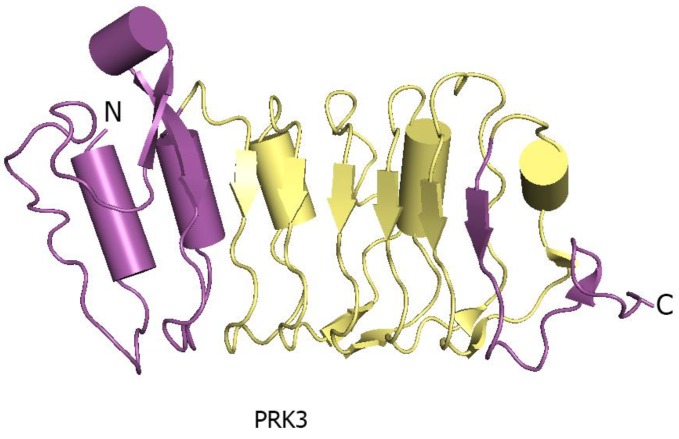
Capped region in the LRR ectodomain: PRK3 (PDB ID: 5WLS) containing a capped domain in both the N- and C-terminal regions of its ectodomain. The PRK3 ectodomain is colored in pale-yellow and the capped regions are colored in magenta color.

**Figure 5:**
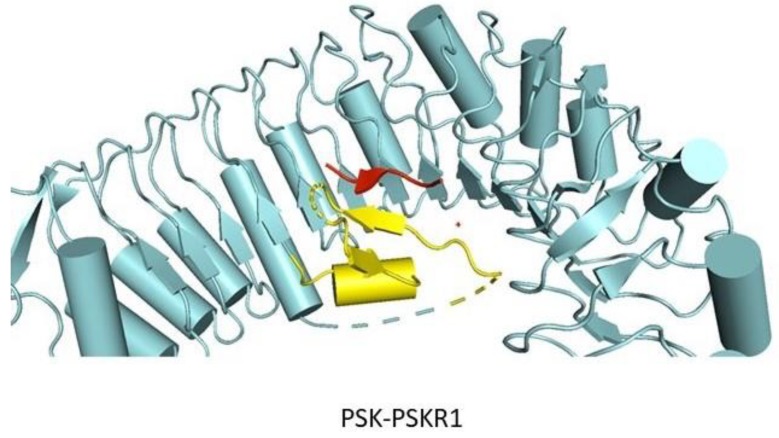
Island domain in the LRR-RK ectodomain. The PSK peptide colored in red interacts with the island domain of PSKR1 (PDB ID: 4Z63) colored in yellow. PSKR1 ectodomain is colored in cyan.

The N- and C-terminal regions of LRR ectodomains contain hydrophilic capped regions that mask the hydrophobic ends from solvent accessibility [19]. The capped areas can also be recognized by the presence of disulfide bonds which help to maintain the overall structure integrity [19]. The well-defined N- and C-terminal capping regions of PRK3 are made up of mostly anti-parallel β sheets (Figure 4) [17]. Capped regions in PRK3 act to shield the hydrophobic core from solvent exposure [19] (Figure 4). Some of the LRR ectodomains like PSKR1 contain a characteristic island region in their structures [7]. An island region or island domain (ID) is a spacer region between two tandemly arranged LRR repeats. In the case of PSKR1, the ID provides an interface for ligand interaction. The ID in PSKR1 also interacts with its ligand PSK. The functionality of the ID is still dubious with further studies necessary to uncover its actual function (Figure 5).

### 3.2. Structural Features

**Figure 6:**
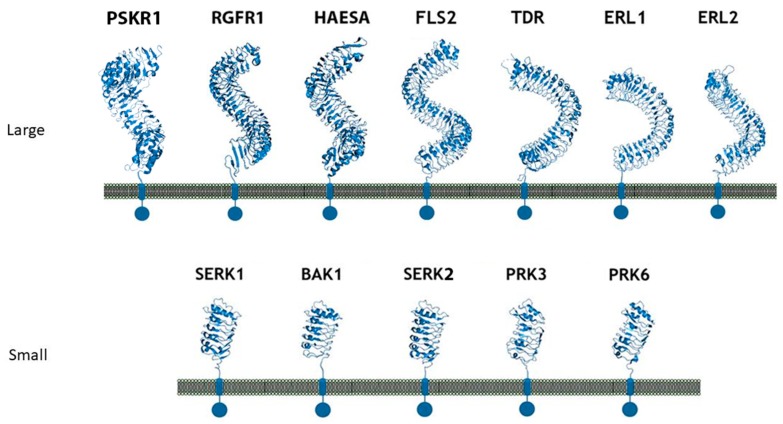
Ribbon diagram depicting the tertiary structures of the large and small LRR-RK ectodomains. The transmembrane domain is shown as blue columns and the kinase domains are denoted as blue spheres. The LRR-RK ectodomain structures vary in shape and size, but the overall architecture is fairly conserved. Top panel (Large): PSKR1 (PDB ID: 4Z63) contains a spiral shaped extracellular domain consisting 27 LRR repeats. It also contains an island domain. RGFR1 (PDB ID 5HYX) is an LRR-RK that binds to a peptide that promotes root meristem growth. HAESA ectodomain (PDB ID 5IXO) interacts with a peptide ligand that is involved in inflorescence. The extracellular domains of FLS2 (PDB ID 4MNA), TDR (PDB ID 5JFK), ERL1 (PDB ID not available), and ERL2 (PDB ID not available) are all composed of more than 20 repeating LRR units. Unlike PSKR1, none of these LRR-RKs contain an island domain. It has been found that the interior concave surface of these LRRs are responsible for ligand/peptide hormone binding. Bottom panel (Small): SERK1 (PDB ID 4LSC), BAK1 (PDB ID 4MN8), and SERK2 (PDB ID 5GQR) all belong to the SERK family of small LRR kinases. The SERK family of LRRs contain five LRR repeats and mostly function as co-receptors. BAK1 is involved in FLS2 mediated signaling, SERK1 acts as a co-receptor in PSK-PSKR1 signaling, and SERK2 takes part in TDIF-TDR/PXY signaling cascade. Both the ectodomains of PRK3 (PDB ID 5WLS) and PRK6 (PDB ID 5Y9W) are made up of six LRRs and belong to the PRK family of small LRR-RKs. The AtLURE1.2 peptide is recognized by PRK6, and the peptide interacts with the C-terminal region of the PRK6 ectodomain.

Many LRR-RK extracellular domains have been structurally characterized. These structures have provided useful information about receptor-ligand interactions, receptor-co-receptor complex formations, and the structural rearrangements that lead to kinase domain activation [15,18,182,183]. Studies have shown that the LRR ectodomains mostly provide a platform for ligand or co-receptor association. Based on their ectodomain structures, plant LRR-RKs can be categorized into two broad groups: (a) the large LRR ectodomain, and (b) small LRR ectodomains (Figure 6). FLS2, TDR, ERL1, PSKR1, CLV1, etc., all belong to the large LRR ectodomain family. FLS2 binds to bacterial flagellin (flg22); PSKR1 recognizes a small post-translationally modified peptide PSK which governs the cell division; TDR perceives a peptide ligand TDIF which promotes procambial cell proliferation and inhibits cell differentiation; and ERL1 interacts with a cysteine-rich peptide EPF1, among others [16,18,184,185,186].

SERK1, SERK2, SERK3/BAK1, and SERK4 from *Arabidopsis thaliana* belong to the small LRR ectodomain family (Figure 6). SERK1, SERK2, and BAK1 have been found to interact with some large LRR ectodomain LRR-RKs. BAK1 acts as a co-receptor in the FLS2 mediated immune response and has also been found to interact with the FLS2 ectodomain [16,187]. SERK1 acts as a binding partner with either PSKR1 or TDR [184,188]. It has also been found that these SERK family members usually function as co-receptors and engage with large LRR ectodomains to facilitate the kinase activation pathway [189]. SERK family members have also been featured as non-ligand binding receptors [187]. Based on these findings, some scientists have hypothesized that the large LRR ectodomains work as the main ligand binding unit, whereas the small LRR ectodomains mostly function as co-receptors [119,186,187,190].

The large LRR extracellular domains usually have 20–29 LRR repeats that form a twisted superhelical structure to bind ligands. The small LRR proteins, in contrast to large LRR ectodomains, contain mostly five LRRs that fold as a small arc-shaped structure (Figure 6). Recently, *Arabidopsis thaliana* Pollen Receptor Kinase 3 (PRK3) and Pollen Receptor Kinase 6 (PRK6) LRR ectodomain structures have been determined with PRK3 resembling closely the SERK family members (SERK1, SERK2, and SERK3/BAK1) and subsequently being grouped into the small LRR ectodomain family [19] (Figure 6). Structurally, PRK6 is similar to PRK3 as they both contain six LRRs with a C shaped conformation. Interestingly, PRK6 binds to a cysteine-rich peptide LURE1.2, the first report to show a small LRR ectodomain acting as a ligand binding unit [54]. As a large set of LRR structures remain to be solved, it may be too early to label the small LRR ectodomains exclusively as co-receptors and non-ligand binding entities.

**Figure 7:**
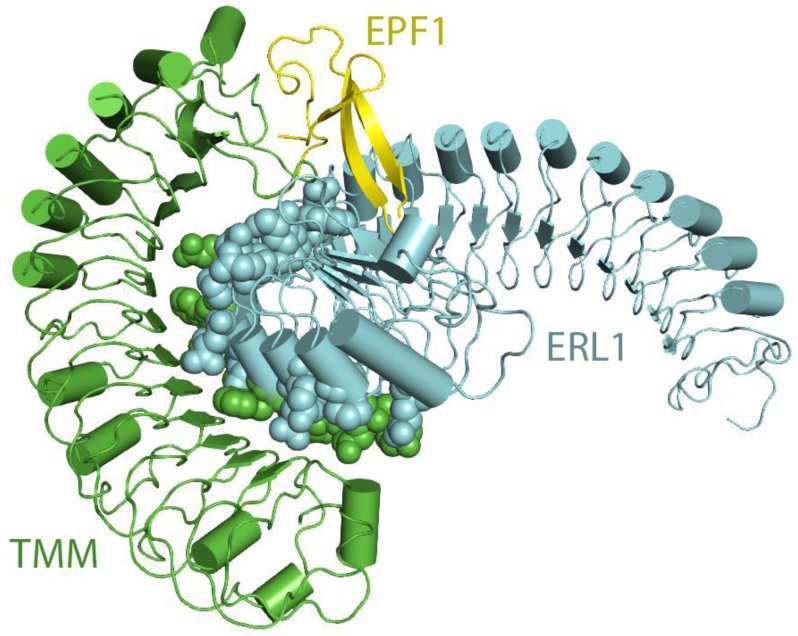
Ribbon diagram showing the interactions between an LRR-RK and LRR-RP. TMM, a typical LRR-RP, that lacks a kinase domain interacting with both the ERL1 ectodomain and an EPF1 peptide and acts as a “Specificity switch.” TMM and ERL1 form a receptor complex that interacts with EPF1 and EPF2, whereas, ERL1 can independently communicate with EPF4 and EPF6. The residues on ERL1 that interact with the TMM ectodomain are shown in cyan colored spheres. The residues on TMM interacting with ERL1 ectodomain are shown in green spheres. ERL1 is colored in cyan, green represents TMM, and EPF1 is depicted in yellow. (PDB files of ERL1, TMM and EPF1 were kindly provided by Dr. Jijie Chai).

Another family of receptor proteins known as LRR-RLP have also been implicated in various plant signaling processes. TMM and CLV2 are two examples of LRR-RLPs. TMM is an integral member of the EPF1-ERf signaling complex regulating stomatal formation. Structurally, the TMM ectodomain has 10 LRRs and interacts with the N-terminal convex region of the ERf ectodomain (Figure 7). TMM can also interact with ERECTA, ERL1, or ERL2 in a ligand-independent manner [185]. Analysis of the TMM’s concave surface has shown that the central portion of the complex is predominantly neutral with positive charges found at the periphery. The interactions between TMM and ERL1 are based on their charge complementarity and van der Waals contacts.

Moreover, TMM is also capable of interacting with EPF1 and EPF2 ligands [185]. CLV2, another LRR-RLP that helps in maintaining the plant stem cell population, has been discovered with no apparent interaction between CLV3 and other CLE peptides [191]. It is possible that similar to TMM, other LRR-RLPs can interact with ligand peptides or small hormones while also acting as co-receptors.

Another striking structural feature of the LRR ectodomain is the ID (Figure 5). The PSKR1 ectodomain interacts with the PSK ligand, leading to heterodimerization of PSKR1 with the SERK1 co-receptor. PSKR1 ID is mainly composed of antiparallel β strands that interact with the N-terminal region of the SERK1 ectodomain [184]. van der Waals forces mostly mediate the interaction between PSKR1 ID and SERK1. The ID is a unique structural feature found, so far, only in BRI1, PSKR1, and RPK2 ectodomains [17,184,192]. RPK2 also harbors two island domains; one located in the N-terminal region and the other in the central region [192]. The function of the RPK2 IDs is still unknown. Furthermore, the ectodomains of TDR, FLS2, PRK3, PRK6, and ERL1 are devoid of ID.

### 3.3. Glycosylation Patterns in LRR Ectodomains

In plants, many secreted proteins, cell surface receptors, as well as membrane and cell wall-associated proteins are frequently modified with complex glycan structures. Two major types of glycosylation can occur: N-linked glycosylation on the amide group of the asparagine residues and O-linked glycosylation on the hydroxyl group of either the serine, threonine, or hydroxylproline residues [193,194,195,196,197,198] In eukaryotes, the *N*-glycosylation process is a conserved and highly essential co/post-translational modification that takes place when proteins follow the secretory pathway inside the endoplasmic reticulum (ER) and Golgi, in which a multi-subunit enzyme and an oligosaccharyl tranferase complex governs the transfer of a core N-glycans (Glc3Man9GlcNac2) to the nascent polypeptide chains [199,200].

*N*-glycosylation happens on specific asparagine residues within the consensus sequence of Asn-X-Ser/Thr (NXS/T motifs) where X can be any amino acid except proline and aspartate [193,194,196]. Typically, *N*-glycans reside on the exposed surface of the protein and form flexible extended branches [201]. *N*-glycosylation ensures not only proper folding of the conjugated proteins but it also plays a significant role in various biological processes such as maintaining protein conformation and stability, executing cellular interactions, and protecting from protease degradation [202,203]. Mammalian TLR3 contains 23 LRRs and possesses 11 N-glycans. These glycans significantly contribute to the molecular mass of the protein and are important for TLR3 mediated downstream signaling pathway [204,205]. So far, plant N-glycans have been classified into two groups: high mannose type and complex type N-glycans. It has been discovered in *Arabidopsis thaliana* that most of the N linked glycans are of complex type [206]. Improper *N*-glycosylation in the ER and Golgi bodies can severely affect the abiotic stress tolerance in *Arabidopsis* [207,208].

**Figure 8:**
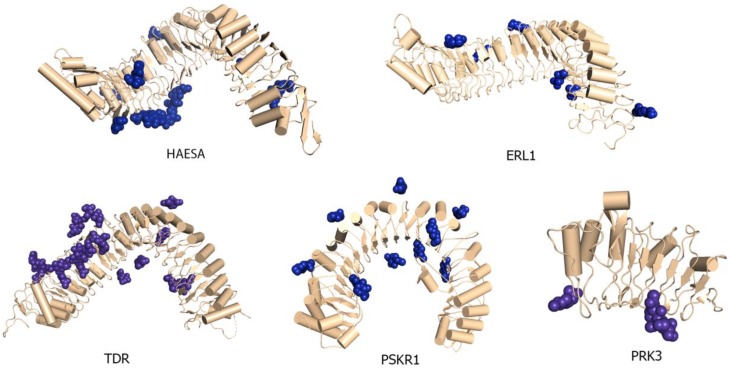
N-Glycosylated LRR ectodomain structures: HAESA (PDB ID: 5IYX), ERL1, TDR (PDB ID 5JFK), PSKR1 (PDB ID: 4Z63), and PRK3 (PDB ID: 5WLS) are all ectodomain structures that contain attached sugar residues. The LRR ectodomains are colored in wheat. The Glc-NAc residue moieties are represented as blue spheres.

Several conserved *N*-glycosylation motifs such as N(X)S/T, have been found on the extracellular domains of the LRR-RKs in *Arabidopsis thaliana*. FLS2, EFR, TDR, PSKR1, BAK1, PRK3, PRK6, ERL1, and MIK1 all contain putative *N*-glycosylation motifs. The expressed recombinant ectodomains of LRR proteins are often larger than their predicted molecular weights, as shown in the recombinant ERECTA ectodomain protein which has a molecular weight (MW) of approximately 70 kD and is about 10 kD larger than its predicted MW of 60 kD. Similar discrepancies in molecular weights have been found in TDR, PRK3, MIK1, etc., indicating that these proteins were likely glycosylated when expressed in a heterologous expression system. The crystal structures of HAESA, ERL1, TDR, PSKR1, and PRK3 ectodomains also contain some N-linked glycans [18,19] (Figure 8). 

In the recombinant MIK1 ectodomain protein, N82, N271, N417, and N535 residues have been found by mass-spectrometry (MS) to be heavily glycosylated (unpublished data, Guozhou Xu). Characterization of these glycosylation moieties will shed light onto their proper function. The Glyco-EP bioinformatics program has predicted the N-linked and O-linked glycosylation sites on the ectodomains of the ERfs and TMM [209] (Table 2).

It has been reported that the putative *N*-glycosylation motifs N(X)S/T are predominantly present within the α-helical region located on the convex side of the LRR domain and are essential for the proper folding of the overall structure [210]. Several studies have revealed the effect of *N*-glycosylation in plant LRR-RK function. In *Arabidopsis*, EFR recognizes bacterial EF-Tu by interacting with a Pathogen Associated Molecular Pattern (PAMP) molecule elf18. It has now been found that *N*-glycosylation is essential for this recognition [211].

When some of the conserved *N*-glycosylation motifs were altered, it was observed that plants became more susceptible to bacterial infection and developed disease symptoms [205]. Based on these observations, it has been suggested that mutations in *N*-glycosylation sites may impede the EFR-elf18 interaction and downstream signaling networks [211]. N-143 and N-288 residing on the outer surface of the EFR ectodomain were found to be essential for elf18 ligand binding, EFR mediated immune responses and protein stability [211]. It has also been suggested that BAK1 and FLS2 are less affected by the alteration of *N*-glycosylation sites [211].

Interestingly, some reports show that under-glycosylated EFR, and non-glycosylated FLS2, both fail to form efficient ligand binding sites [211]. Mutating the glycosylation sites of tomato Cf9 affects its downstream activity of avirulence factor 9 mediated programmed cell death [210]. However, these mutations do not affect Cf9 protein stability. Mutational analyses have also revealed four essential glycosylation sites on the convex side of the Cf9 LRR ectodomain [210]. These results indicate the importance of *N*-glycosylation in LRR domain folding and stability. Furthermore, it is evident that N-glycans significantly contribute to LRR-Ligand binding activity, function, and the elicited downstream signaling network.

## 4. Mechanism of Ligand Perception by LRR Ectodomain

### 4.1. LRR Ectodomains Interaction with Small Secreted Peptide Ligands

**Figure 9:**
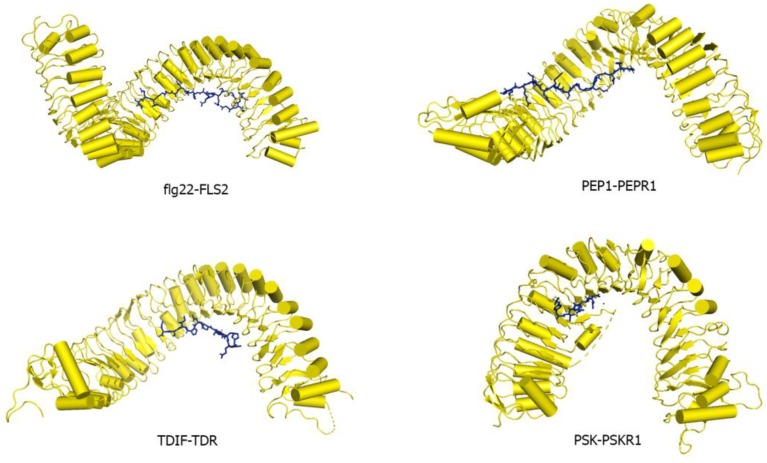
LRR ectodomains interacting with small post-translationally modified peptides: flg22 epitope interacting with FLS2 ectodomain (PDB ID 4MNA); PEPR1 ectodomain (PDB ID 5GR8) is responsible for interacting with pep1 peptide; TDIF peptide interacts with TDR (PDB ID 5JFK); and PSK peptide binding on the island domain of PSKR1 ectodomain (PDB ID: 4Z63). The peptide ligands are denoted in blue stick representation and the LRR ectodomains are represented in yellow.

FLS2 is an LRR-RK that senses a 22 amino acid long conserved epitope of bacterial flagellin flg22. It is the first reported structure that provides critical biophysical and biochemical information of a peptide ligand interacting with a plant LRR-RK [15,16,212]. FLS2 has a large ectodomain composed of 29 individual LRRs with no ID present in the ectodomain. The flg22 peptide binds on the concave inner surface of the FLS2 extracellular domain (Figure 9). flg22 contacts LRRs 3–18 of FLS2 allowing various side-chain and main-chain interactions to be observed. The flg22 N terminus is slightly hydrophilic, while its C-terminus is made of mainly small hydrophobic residues [16]. For Flg22 ligand, Gln65, Arg66, Arg72, Ile73, Asn74, Lys77 residues have poor side chain density. Amino acids from Gln65-Ile85 are visible in the density at the backbone positions.

*Arabidopsis thaliana* Pep1 is a 23 amino acid long peptide produced in response to pathogenic attacks and is perceived by the LRR-RKs PEPR1 and 2 [30,213,214,215]. PEPR1 consists of 27 canonical LRRs and is similar to FLS2, TDR, ERL1, and PEPR1 as it lacks an ID [216]. AtPep1 follows the same binding mode of flg22 as it binds to the inner concave surface of the PEPR1 ectodomain, covering LRRs 4–18 [216] (Figure 9). However, the C-terminal interacting region of AtPep1 is considerably different from flg22. The alanine present in the C-terminal portion of flg22 seems to be completely disordered in the FLS2-flg22 complex structure, whereas, the asparagine 23 and the carboxyl-terminal region of AtPep1 forms hydrogen bonding with an arginine residue in LRR 18 of the PEPR1 [16,216]. This arginine forms a specific pocket that helps to lodge the AtPep1 C-terminus. When this invariant C-terminal asparagine is deleted, the binding of AtPep1 to its receptor is lost [216]. In this crystal structure, AtPep1, from residues Lys7-Asn23, Lys7, Lys11, Lys13, has poor side chain electron density.

The HAESA ectodomain interacts with IDA peptides and IDA adopts a fully extended structural arrangement [133]. HAESA ectodomain LRRs 8–10 forms a specific groove that actually fits the Hydorxyproline (Hyp) residue 64 on IDA [133]. This Hyp64 of IDA interacts with a conserved Glu266 residue on HAESA by forming a hydrogen bond. Hydrophobic interactions and hydrogen bonding network contribute significantly to the binding interaction between IDA and HAESA [133]. Interestingly, HAESA can recognize IDL1 peptide, implying that the IDA and IDL family of peptides may use a similar binding pattern [127]. HAESA can differentiate between different dodecamer peptides that are separate from IDA or IDL peptides, like CLV3 and interestingly, SERK1 can interact with HAESA [42,133,217]. Furthermore, SERK1 LRR forms a stable heterodimeric complex with HAESA ectodomain when IDA is present [133,217]. It is also shown that the presence of SERK1 actually increases the binding affinity between IDA and HAESA. Interestingly, the IDA peptide itself also strongly contributes to the interaction between HAESA and SERK1 ectodomains [133]. Therefore, it can be said that SERK1 actually acts as a co-receptor in HAESA mediated specific peptide hormone recognition. In the case of HAESA-IDA crystal structure, amino acids Pro58, lys66, and Arg67 on the IDA peptide have low side chain electron density.

In *Arabidopsis*, TDR and its ligand TDIF are involved in the maintenance of the procambial cells. TDIF regulates the proliferation of procambial cells and inhibits their differentiation into xylem cells [45]. TDIF/CLE41 peptide is 12 residues long, containing hydroxyproline residues and a C-terminal asparagine. Structural and biochemical data indicates that the interactions between the ligand and the TDR receptor at the C-terminal anchoring site provides conserved binding. The N-terminal binding site dictates the differential binding specificities between CLE ligands and the receptor ectodomains [18] (Figure 9). The TDIF-TDR interaction is highly similar to that of HAESA-IDA [133,218]. HAESA ectodomain is made up of 21 individual LRRs. IDA interacts with the LRRs ranging from 2–14 and contains a hydroxyproline residue that forms a hydrogen-bonding network with the LRRs [133]. The IDA hydroxyproline and HAESA interaction seem to be critical for the binding [133]. It can be observed that despite their differences in functionality, TDR/PXY and HAESA ectodomains are similar in shape, size, and conformation; however, they do not share high sequence homology. In the TDR-TDIF structure, the ligand has all side chains and modifications shown up in the electron density. The refinement statistics of the LRR structures are shown in Table 3.

**Figure 10:**
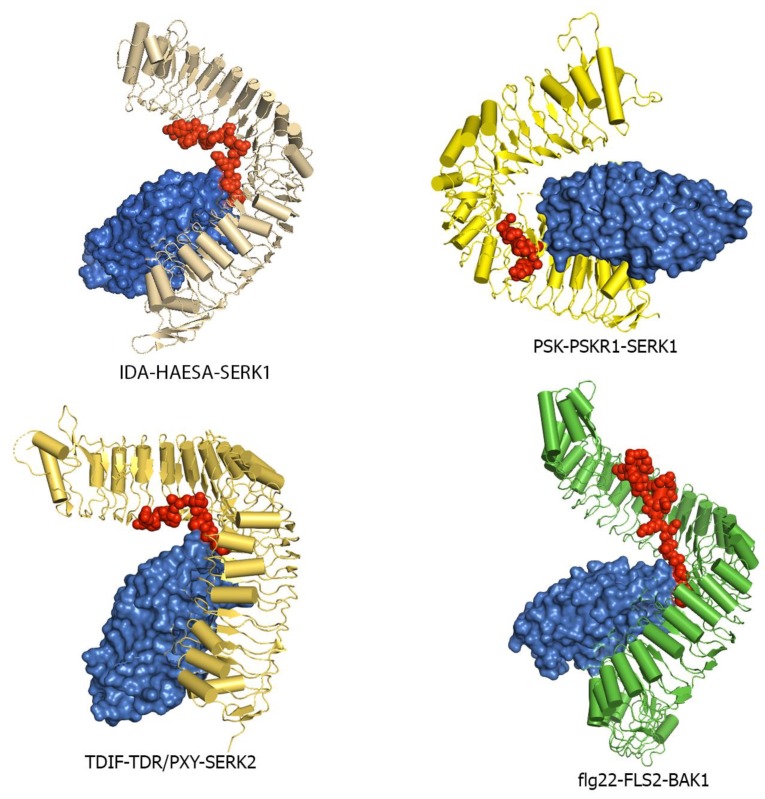
Universal co-receptors: SERK family of LRR-RKs, SERK1 (PDB ID 4LSC), SERK2 (PDB ID 5GQR), BAK1/SERK3 (PDB ID 4MN8), and SERK4 have been found to act as co-receptors for large LRR-RKs. Here SERK1, SERK2, and BAK1 ectodomains (shown in slate colored surface representation) are interacting with the C-terminal region of the HAESA (PDB ID 5IYX), PSKR1 (PDB ID 4Z63), TDR/PXY (5GQR), and FLS2 (4MNA) ectodomains respectively. The peptide ligands are shown in red spheres. The large LRR ectodomain structures are shown via cartoon representation.

The interaction between PSK and PSKR1 ectodomain is fairly strong with an estimated Kd of 7.7 nM in Arabidopsis [184]. PSK adopts an anti-parallel β-sheet conformation while interacting with PSKR1 ID region (Figure 5 and Figure 9). Various non-covalent interactions like hydrogen bonding, van der Waals forces play significant roles in PSK-PSKR1 LRR interaction, whereas the sulfate groups of PSK directly interact with PSKR1. It was found that binding of PSK does not induce oligomerization of the PSKR1 receptor ectodomain and it was also found that PSKR1 ectodomain is capable of interacting with the SERK family of co-receptors [184]. The crystal structure of the PSK–PSKR1 ectodomain in complex with SERK1 ectodomain has been determined [184] (Figure 10). Structural studies of Wang et al. have suggested that the PSK ligand can enhance PSKR1-SERK1 heterodimerization and thereby induce the trans-phosphorylation reaction of the respective kinase domains [184]. The PSK ligand has all side chains and modifications shown up in the electron density.

In Arabidopsis, five LRR-RKs have been identified that recognize RGF peptides and actively control the root meristem development process [120]. It has been found that RGF1 interacts with RGFR1 LRR-RK very strongly, as the interaction between RGF1 and RGFR1 suffers considerably when a non-sulfated RGF1 peptide is used, demonstrating the importance of sulfation in RGF and RGFR recognition [118]. It has been found that the van der Waals interaction between the sulfate group and RGFR1 Gly220 further strengthens their complex formation [118]. The binding mode of RGF1 and RGFR1 is similar to AtPEP1 and PEPR1 [216]. The RXR motif of RGFR1 plays a crucial role in identifying the RGF1 peptide and the motif interacts with the last residue Asn of RGF1 peptide [118]. Based on the structural architecture, the N- and C-terminal region of RGF1 interacts with positively charged surface of RGFR1 and the central region of RGF1 peptide fits into the negatively charged region of the receptor ectodomain [118]. Furthermore, the SERK family of LRR-RKs have been found to be important for RGF mediated signaling cascade. It is suggested that SERK1/2/BAK1 can act as co-receptors for RGF receptors [118]. In the RGF1-RGFR1 crystal structure, the peptide ligand has decent electron density and out of 13 residues, all the residues are clearly visible except Arg11 where the side chain has low electron density (Table 3).

Recent structural studies have determined that LRR-RLPs also play critical roles in ligand-receptor interactions [185]. It has been found that certain LRR-RLPs work as a specificity switch for ligand perception. Structural investigations have found that TMM forms constitutive complexes with either ER or ERL1 (ERfs) that are able to recognize the cysteine-rich peptides EPF1 and EPF2. It has been noted that ERfs cannot independently interact with EPF1 and EPF2. Structural elucidation has revealed that the interactions between TMM and ERL1 create a binding pocket that perceives EPF1 and EPF2, thereby showing that the TMM-ERf complex as a whole acts as a receptor for ligand binding [185] (Figure 7). However, ERfs can independently perceive EPFL4 or 6, and it does not require TMM. Based on these pieces of evidence it can be suggested that TMM has evolved to provide ligand specificity and to regulate stomatal development [185].

### 4.2. Interactions between CRP and LRR-RK Ectodomains

**Figure 11:**
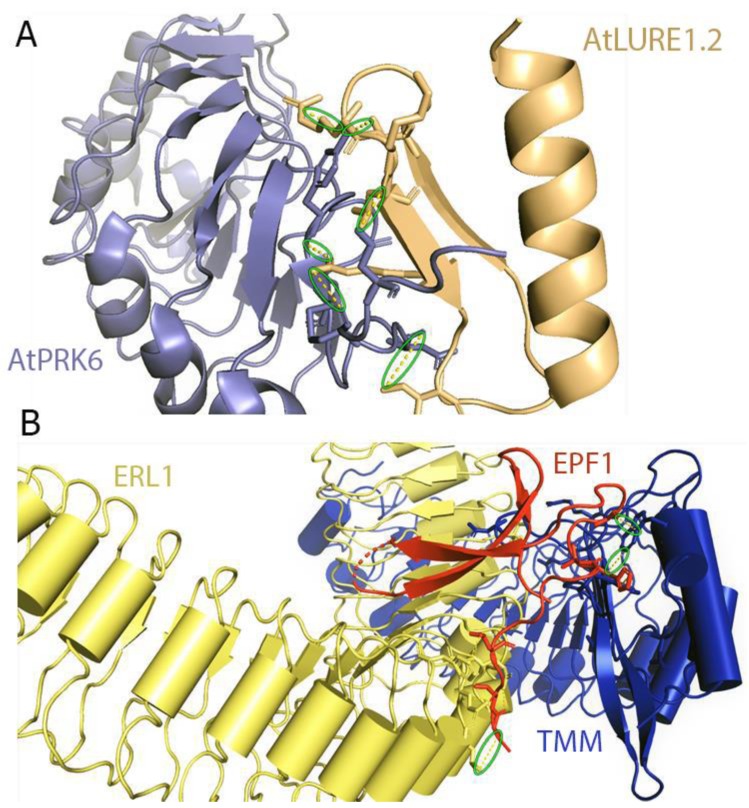
Interactions between CRPs and LRR-RK ectodomains: (**A**) Ribbon diagram showing the binding interface between AtPRK6 and AtLURE1.2 (PDB ID 5Y9W). AtLURE1.2 is a defensin-like CRP that interacts with AtPRK6 at the C-terminal region. AtPRK6 is colored in cyan and AtLURE1.2 is colored in wheat. The interacting residues are represented in stick form whereas the hydrogen bonds, van der Waals, and polar interactions are shown as dotted yellow lines. (**B**) Ribbon diagram showing the interaction interfaces between EPF1, ERL1, and TMM. EPF1 ligand is colored in red with the disordered loop shown as a dotted line, ERL1 ectodomain is colored in yellow, and TMM is colored in blue. The interacting residues are depicted in stick model, whereas the interactions are represented by a yellow dotted line. (PDB files of ERL1, TMM and EPF1 were kindly provided by Dr. Jijie Chai).

Recently, an AtPRK6 ectodomain structure in complex with AtLURE1.2 peptide has been solved (Figure 11A). PRK6 contains six LRRs in its ectodomain adopting a solenoid structure. A capping region shields the hydrophobic residues of the N-terminal region. The C-terminal region of PRK6 contains a disulfide bond [54]. The C-terminal region on the LRR of the AtPRK6 is mainly responsible for interacting with AtLURE1.2. The loop region of PRK6 binds directly to the positively charged surface of AtLURE1.2 peptide [54]. The disulfide bond formed by Cys237 and Cys229 is involved in the interactions between AtPRK6 and AtLURE1.2. Two significant interactions can be observed between PRK6 and AtLURE1.2 concerning shape and charge complementarities. The residues of AtLURE1.2 that are interacting with AtPRK6 are highly conserved in other LURE peptides. Surprisingly, the residues of AtPRK6 that contact AtLURE1.2 are not conserved in other PRK members [54].

Lin et al. have shown that the ectodomains of ERfs can form a stable complex with the TMM ectodomain and the complex functions as the receptor of EPF1, EPF2, or EPFL9. Interestingly, ERfs can interact with EPF1, EPF2, and EPF9 only in the presence of TMM. In contrast, EPFL4 and EPFL6 can easily be recognized by the ERfs in the absence of TMM. However, it was noted that TMM when present, can interfere with the interactions of EPFL4, EPFL6 with ERfs [185]. These results are consistent with the previous findings [219,220]. It can therefore be said that TMM acts as a selective modulator that permits ERfs to distinguish between different sets of ligands. ERf-EPF interactions do not result in significant structural rearrangements or homodimerization in the presence or absence of TMM (Figure 11B). Furthermore, recently it has been found that the SERK family of LRR-RKs can act as coreceptors in ERf mediated signaling pathways regulating stomatal patterning [221]. It is quite possible that co-receptors are required for the ligand-mediated activation of ERfs with SERK kinases playing a significant role in this pathway.

## 5. Ligand-Induced Kinase Activation

Membrane integral receptor kinases (RKs) play essential roles in mediating intercellular communications in most multicellular organisms. The binding of ligands to their extracellular domain(s) usually induces activation of RKs, which triggers either oligomerization or conformational changes of the RKs, their intracellular kinase domains are then activated to initiate signal transduction into the cells [4,5,222,223]. Receptor kinases contain an extracellular domain (ECD) that is usually a ligand-binding domain, a single-helix transmembrane domain (TM), and a cytoplasmic region with a kinase domain (KD). Some RKs have a short extracellular juxta-membrane domain (eJM) between the ECD and TM, and an intracellular JM between the TM and the KD. Following the kinase domain, there is a C-terminal tail (CT) that often provides autophosphorylation sites for the activated KD. Some integral membrane RKs exist as monomers, and ligand binding usually induces homodimerization or heterodimerization with a co-receptor, eventually triggering KD trans-autophosphorylation and activation [4,223,224]. Other RKs form dimers in the absence of a ligand where ligand binding may rearrange the domain conformations of the RK leading to KD trans-autophosphorylation and activation [225]. Both the ECD and the KD may mediate either the preformed or ligand induced RK dimerization. The TMs, JMs, and CTs can also contribute to RK activation. The CT of some RKs contains Serine, Threonine, or Tyrosine sites that are autophosphorylated by the activated KD. The phosphorylated sites on CT provide docking sites for downstream phospho-Ser, Thr, or Tyr binding domains containing signaling proteins to elicit signal transduction [226]. In plants, the activation of LRR-RKs by ligand binding usually requires heterodimerization with a co-receptor. The SERK family of small LRR-RKs have been shown to function as co-receptors to mediate LRR-RKs heterodimerization and activation upon ligand binding [16,187,188,216].

Gene duplication and functional divergence has created the SERK family of small LRR-RKs [7,56]. So far, five SERK protein kinases have been identified in the *Arabidopsis* genome [7]. The SERK family members are highly homologous with similar functions. The SERK members take part in multiple signaling pathways to regulate plant growth and development, but their overall operational methods are still unknown. Multiple structural and biochemical studies have pointed out that SERK family members act as co-receptors and interact with both the ligand and the large LRR ectodomain kinases [16,17,187,188] (Figure 10). The residues on SERKs that are responsible for the large ectodomain interactions are conserved among all the SERK proteins implying that all SERKs can form these complexes [227]. Studies have also shown that ligand binding does not induce homodimerization or cause any major conformational changes to the large ligand binding ectodomain [16,182,184,187,188]. It has been evident that for receptor kinase activation, in addition to the ligand binding, an additional protein is also required. Based on genetic, cellular, biochemical, and structural studies, it can be said that the SERK family of small LRR-RKs act as the additional protein required. SERK family members act as co-receptors to form a complex with the large LRR-ectodomains that cause the architectural remodeling of the overall structure to initiate kinase activation.

Kinase domains of large LRR receptors can physically interact with BAK1, and the SERK family of co-receptors, but their exact structural configuration remains elusive. The kinase domain interaction, therefore, proves the notion of ligand-induced heterodimerization mediated RK activation in plants. SERK family members also act as co-receptors for PSKR1 and TDR, etc. A similar kind of co-receptor binding site is observed in the PSKR1-SERK1 ectodomain complex. The PSK ligand promotes PSKR1-SERK1 interaction, thereby supporting the ligand-mediated heterodimerization model. Surprisingly, it has been found that PSK does not act as the “Molecular Glue” in this scenario; instead, the principal function of PSK is to stabilize the PSKR ID region, which in turn interacts with SERK1 by forming a stable heterodimeric complex [184,186]. This mode of action has been termed as the allosteric activation of PSKR1. It has been hypothesized that PSK induces the complex formation which can lead to trans-phosphorylation of respective kinase domains [184].

The flg22-FLS2-BAK1 structure involves the only heterodimerization with no higher order oligomerization event observed [216]. This again supports the idea that ligand-induced heterodimerization is essential for receptor kinase activation in plants. SERK1, SERK2, and SERK3/BAK1 all interact with TDR/PXY in a TDIF dependent manner [188] (Figure 10). Following the biochemical data, phenotypes observed from SERK triple mutants suggest that SERKs serve as co-receptors of TDR/PXY [54]. It is evident from the crystal structure that the binding of SERK2 does not induce any structural rearrangements [186].

Based on multiple studies, it has been hypothesized that ligand-induced heterodimerization with a co-receptor is a crucial mechanism for the intracellular kinase activation in plants. Studies on plant LRR-RKs have shown that various ligands can strongly promote the interaction between the ectodomain of a receptor and its co-receptor. These strong interactions result in the formation of a heterodimeric complex that brings the transmembrane and kinase domains of the corresponding receptor-co-receptor together. The kinase domains of these RKs go on to interact and form a dimeric conformation for their activation [182,228].

## 6. Conclusions

LRR containing proteins have been identified in archaea, bacteria, eukaryotes and even in viruses [171]. LRR proteins actively partake in cellular signaling, cell adhesion, DNA repair, transcription, RNA processing etc. [172,180,229]. Most of these proteins are involved in protein-protein, protein-ligand, protein-hormone interactions [230]. It has also been found that LRR proteins are capable of interacting with non-protein ligands, such as an LRR protein CD14 can recognize and directly communicate with bacterial lipopolysaccharide (LPS) [172]. Based on the different lengths of the LRR motifs and consensus sequence, LRR proteins are classified into seven major classes: Ribonuclease Inhibitor (RI) type, cysteine-containing (CC), bacterial, SDS22-like, plant-specific, typical, and TpLRR [231,232,233,234,235,236].

The evolutionary history of LRR proteins is still unknown. Kobe and Deisenhofer predicted that LRR proteins might have a bacterial origin while also hypothesizing that LRR proteins may have evolved before the eukaryotes even appeared [171,229]. However, the possibility of horizontal gene transfer (HGT) cannot be ruled out, where bacteria may have acquired the LRR proteins from eukaryotes. Based on sequence conservation within each class, Kajava suggested the independent originations of different groups of LRRs [181]; whereas, Andrade et al. have a contrasting viewpoint; based on their homology-based study hypothesizing that that LRR proteins have a common ancestor [237]. We do not know about the evolutionary force that was driving the evolution of each LRR protein subfamily, and what was the function of the LRR proteins during each stage of development. Subsequently, in the case of LRR protein evolution, a lot of unanswered questions remain.

It is well known that plant LRR-RKs regulate a variety of cellular processes inside plant cells. LRR-RKs control multiple signal transduction pathways leading to cell division, cell differentiation, stem cell balance, immune and pathogen response, symbiosis, plant development, pollen tube guidance, and ovule targeting [1,12,13,25,37,48,70,71,87,105,142,167,169]. Various cellular, biochemical, genetic, and structural biology studies have provided insights into their structure, function, and mechanism of kinase activation. It is especially worth mentioning that structural biology approaches have provided detailed molecular and mechanistic insights about the LRR-RK ectodomain architecture and ligand binding modes [16,19,54,182,183,184,187,212,216,218]. Multiple structural and functional studies have successfully elucidated the LRR-RK receptor-co-receptor interactions, ligand-mediated heterodimerization, structural rearrangements, and kinase domain activation. The LRR-RKs control two major functions in the plant body: plant development and growth and defense against pathogens [238]. It is evident from various studies that these receptor kinases contain varying numbers of LRRs in their ectodomains. We firmly believe that this multiplicity allows them to explicitly recognize their cognate ligands, peptide hormones, and co-receptors [228].

Although the overall LRR-RK structures are relatively similar, their corresponding ligands are very much diverse. Structures of LRR-RKs in complex with small peptides, CRPs, and other small molecules have been described in much detail with several structural models having clearly shown that ligand binding induces the kinase domain activation to start the subsequent downstream signaling cascade. It has been frequently observed that ligand binding induces a heterodimerization event with a small co-receptor kinase or an LRR-RLP which is devoid of a kinase domain. However, our understanding of plant RK mediated signaling is still limited with many questions remaining to be addressed.

At first, it is unclear whether the SERK family members only function as the universal co-receptors or whether they have any other intrinsic functions. It is possible that some other proteins can also act as co-receptors and mediate receptor kinase activation. Previously, we have reported that AtPRK3 is structurally similar to the SERK family members. Based on structural similarity, sequence conservation, and a conserved surface patch on the surface of AtPRK3, we hypothesized it to serve as a co-receptor [19]. It is possible that some PRK family members can work as co-receptors for other PRKs or MIK1, MIK2, and MIDS1 in pollen tube development. It is important to decipher how AtPRK3 partake in ovule targeting and find its interacting partners.

Moreover, AtPRK3 and AtPRK6 share more than 60% sequence identity, and only AtPRK6 can recognize the AtLURE1.2 peptide. It will be interesting to address the question in relation to AtPRK3 being unable to interact with AtLURE1.2. Is there any other unidentified ligand present that interacts with AtPRK3 or can AtPRK3 only act as a co-receptor? Secondly, as for the functionality of LRR-RLPs in receptor kinase signaling, so far, it has only been demonstrated that TMM acts as a specificity switch to recognize the correct ligands [185]. It is still unclear how other LRR-RLPs contribute to ligand binding and heterodimerization activities. Our knowledge about signaling crosstalk is also limited.

Cellular and genetic studies on TDR, CLV1, and ERECTA have revealed that the downstream signaling components are diverse. However, there is very scarce information about the downstream signaling components available so far [13,14,44,182,239,240]. We still know little about how structurally similar kinase domains can activate different downstream pathways. Very few direct downstream signaling components of plant RKs have been discovered so far with very little information available on how the signal propagates inside the cell. Besides, there are still many ligand-receptor pairs that remain to be discovered, and structural characterization is further required to understand their mechanism of action. Biochemical, genetic, cellular, structural, and proteomics studies need to be convened to identify more ligands and downstream components of LRR-RK signaling. Integration of the existing structural models with cellular and genetic studies will provide a better platform to investigate the plant membrane-bound receptor-mediated signaling cascades further. Future structural determination of the full-length LRR-RKs will be another vital step to understand the mechanism of RK activation fully. It is challenging to crystallize the full-length LRR-RKs due to the inherent flexibility between the ectodomain and kinase domain. Recent advancement in cryo-Electron Microscopy (cryo-EM) has provided a better approach in this matter. So far, it has been suggested that ligand-induced receptor heterodimerization is probably the preferred mechanism for plant RK activation. However, we only focus on the structural studies of the LRR-RKs ectodomains and the peptide ligands they perceive. There is a large variety of RKs present in plants. Further investigation of their structure and mechanism of ligand perception will help provide a complete understanding of plant RK activation.

In recent years, multiple LRR-RK ectodomain structures have been determined, but studies on the structures of their KDs are lacking. It is widely considered that the KDs are relatively conserved and contain 12 subdomains that fold into a two-lobed structure [241,242]. Phylogenetic relationships among the KDs and LRR motif arrangements were used for the classification of the LRR-RK genes; in *Arabidopsis thaliana*, LRR-RKs have been classified into 12 subgroups. Five subgroups are present in *Oryza sativa*, and 14 subgroups in *Populus trichocarpa* [2,243,244]. From an evolutionary perspective, the origin of LRR-RK genes from diverse plant species including algae, bryophytes, angiosperms, vascular plants, among others, are not well understood. It is also not known how the exon/intron structures and protein sequences have impacted the evolution of LRR-RKs.

Substantial available genetic, phylogenetic, cellular, and structural information about the LRR-RKs has improved our knowledge about LRR-RK evolution, ligand perception, and plant signaling. Further studies are still required to understand the evolution, cellular, molecular, and mechanistic basis of the LRR-RKs mediated plant signaling pathways as a whole. A unified theory based on this information will pave our way to fully understanding plant growth, development, and their interactions with the environment.

## Figures and Tables

**Table 1:** Expression, functions, peptide ligands, UniProt IDs, and Protein Data Bank(PDB) IDs of the major known receptor kinases (LRR-RKs) in *Arabidopsis thaliana.*

LRR Name	Peptide Ligands	Expression	Function	UniProt ID	PDB ID
ERECT	EPF1/2 EPFL4/5/6	Shoot Apical Meristem (SAM), Xylem, Phloem, Epidermis [20,21]	Stomatal patterning, regulates inflorescence [22,23,24,25]	Q42371	X
ERL1	EPF1/2	Developing organ, bud clusters, flowers, SAM, leaf primordia [26]	Stomatal patterning, inflorescence, organ shape [25,26,27]	C0LGW6	X
ERL2		Developing organ, bud clusters, flowers, SAM, leaf primordia [26]	Stomatal patterning, inflorescence, organ shape [25,26]	Q6XAT2	X
PEPR1	PEP1	Leaf, flower, root [28,29]	Pathogen-associated Molecular Pattern (PAMP)-triggered immune signaling [30,31]	Q9SSL9	5GR8
PEPR2		Leaf, flower, root	Immune signaling similar to PEPR1	Q9FZ59	X
PSKR1	PSK	Root primordia, leaves, stems and flowers [32,33,34]	Plant cell differentiation, somatic embryogenesis, cellular proliferation, plant growth [35,36]	Q9ZVR7	4Z63/4Z64
PSYR1	PSY1	SAM and elongation zone of root meristem [37]	Cellular proliferation and plant growth, not involved in Phytosulfokine (PSK) sensing [35,36,37].	Q9C7S5	X
EFR		Leaf protoplast, petal, stigma [38]	Involved in plant defense [39,40]	C0LGT6	X
PXC2		Cotyledons, shoot apex, hypocotyls [41]	Vascular development [41]	Q9LZV7	X
CLV1	CLV3 CLE2	SAM and early flower meristem [42,43]	Meristem maintenance [42,43]	Q9SYQ8	X
TDR	TDIF	Vascular meristem [44]	Regulation of procambium maintenance [44,45,46]	Q9FII5	5JFK/5GIJ 5GQR
MIK1	LURE1.2 (?)	Pollen tubes [46]	Pollen tube guidance, vascular tissue development [46]	Q9M0G7	X
BAME1	CLV3 CLE5/11/18/19/22/26/40/41/42	Roots, leaves, flowers, anther, and gynoecium [47,48]	Gametophyte development, anther development, vascular strand development within leaves [47,48,49]	O49545	X
BAME2	CLV3 CLE11/18/19/22/25/40/41/42	Seedlings, roots, flowers, cortex of developing stems, anther, and gynoecium [47]	Male gametophyte development, ovule development, vascular strand development within leaves [47,48]	Q9M2Z1	X
FLS2	flg22	Ubiquitously expressed [15]	Flagellin perception, innate immune response [15,50]	Q9FL28	4MNA/4MN8
PRK3		Pollen and flowers [51]	Pollen tube growth, pollen germination [52]	Q9M1L7	5WLS
PRK6	AtLURE1.2	Tip of the pollen tube [53]	Pollen tube guidance, LURE perception [53,54]	Q3E991	5Y9W/5YAH
BAK1		Ubiquitously expressed [55]	Brassinosteroid signaling, PAMP mediated immune response, interacts with FLS2, Elongation Factor tu receptor (EFR). Involved in Phosphorylation of BRI1.	Q94F62	3ULZ
SERK1		Vascular bundles, flowers, and embryo sac [56,57]	Phosphorylation of BRI1, brassinolide signaling pathway [58]	Q94AG2	4LSC
SERK2		Developing vascular tissues, flowers, embryo sac [58]	Brassinosteroid dependent and independent pathways, male gametophytic production [58]	Q9XIC7	5GQR/4Z61
SBIR1		Mostly in leaves and flowers [59]	Activation of plant defense and cell death [60]	Q9SKB2	X
PXL1		Mostly in root	Procambium maintenance during vascular tissue maintenance	Q9FRS6	X
EMS1		Young buds, flowers, organ primordia, young pollen grains [61]	Embryonic development. In seeds plays a role in cell size enhancement	Q9LYN8	X
FEI1		Root meristem and elongation zone	Regulates cell wall function, cellulose biosynthesis [62]	C0LGF4	X
GSO1	CIFs	Seeds, developing embryos, flower buds, and roots [63,64]	Embryo and cotyledons epidermal surface development regulate root growth [63,65]	C0LGQ5	X

X denotes no structure is available in the PDB. (?) denotes debatable.

**Table 2:** Predicted glycosylation sites in the ectodomains of ERECTA family of LRR domain-containing transmembrane receptors (ERfs), TMM and the mature Epidermal Patterning Factors Like (EPFLs).

	N-Linked Glycosylated Sites	O-Linked Glycosylated Sites
**ERECTA-ECD**	N65, N74, N203, N221, N234, N305, N329, N409, N457, N510, N528, N543	T48, T225, S570
**ERL1-ECD**	N68, N77, N226, N237, N308, N332, N377, N412, N441, N460, N532, N537, N547	T156, T164
**ERL2-ECD**	N70, N79, N228, N239, N310, N334, N379, N414, N443, N462, N469, N534, N539, N549	T158, T166, T254, T551, T576
**TMM-ECD**	N181, N196, N362	T25, T28, T46, T48, T112, T116, T242, T250, T312, T316, T388, T432, T440, T441, T448, T465, T468, T469, T470, T471
**mEPF1**	N98	S55
**mEPF2**		T69, S71, S72, S77, S118
**mEPF9/Stomagen**		S61, T62, T65, T67

**Table 3:** Crystal structure refinement statistics of different LRR-RK and ligand complexes.

PDB ID	5IXT (HAESA-IDA)	4MN8 (FLS2-flg22)	4Z63 (PSKR1-PSK)	5GR8 (PEPR1-AtPEP1)	5JFI (TDR-TDIF)	5Y9W (AtPRK6-AtLURE1.2)	5HYX (RGFR1-RGF1)
Space group	P 3_1_ 2 1	P 3_2_ 2 1	P 4_3_ 2 2	P 1 2_1_ 1	P 4_1_	C 1 2 1	H 3
Resolution (Å)	1.86	4.0	2.51	2.59	2.75	1.85	2.6
Clash score (percentile)	1	15	6	9	18	8	6
R_work_	17.9	24.9	22.9	23.4	21.4	20.4	19.1
R_free_	20.8	27.9	27.6	28.8	26.7	24.1	23.3
Completeness	99.8% (98.6%)	99.2% (99.7%)	98.4% (99.6%)	99.2% (89.7%)	98.9% (88.5%)	98.2% (97.7%)	98.8% (100%)
RMSD bond (°)	1.48	0.889	1.445	1.642	1.211	1.08	1.421
RMSD Length (Å)	0.008	0.003	0.008	0.009	0.006	0.007	0.008
Wilson B factor (Å^2^)	80.0	87.7	37.2	58.3	59.6	28.5	53.4
Ramachandran Favored	X	90.7%	96.6%	95.58%	86.11%	X	96.51%
Ramachandran Allowed	X	9.01%	3.3%	3.68%	11.84%	X	3.33%
Ramachandran Outliers	X	0.32%	0.1%	0.35%	2.06%	X	0.16%

X denotes no information is available.
